# 2018 Audited schedule of changes in net assets

**DOI:** 10.5195/jmla.2019.821

**Published:** 2019-10-01

**Authors:** BKD

**Affiliations:** CPAs and Advisors, on behalf of the Medical Library Association, kelly.weaver@mci-group.com, Chicago, IL

The table below summarizes the association’s financial status as of December 31, 2018. For a more complete audit report and related information, see the Audited Financial Statements 2001–2018 (members only). This report includes balance sheets, fund status reports, budgeted and actual revenues and expenditures, and a schedule of investments. Members may obtain a copy of the audit report from MLA headquarters.

**Table 1:**
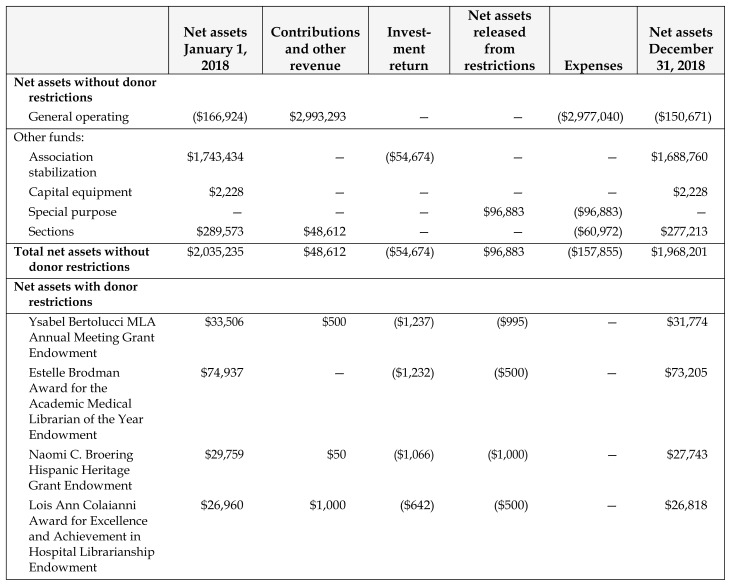

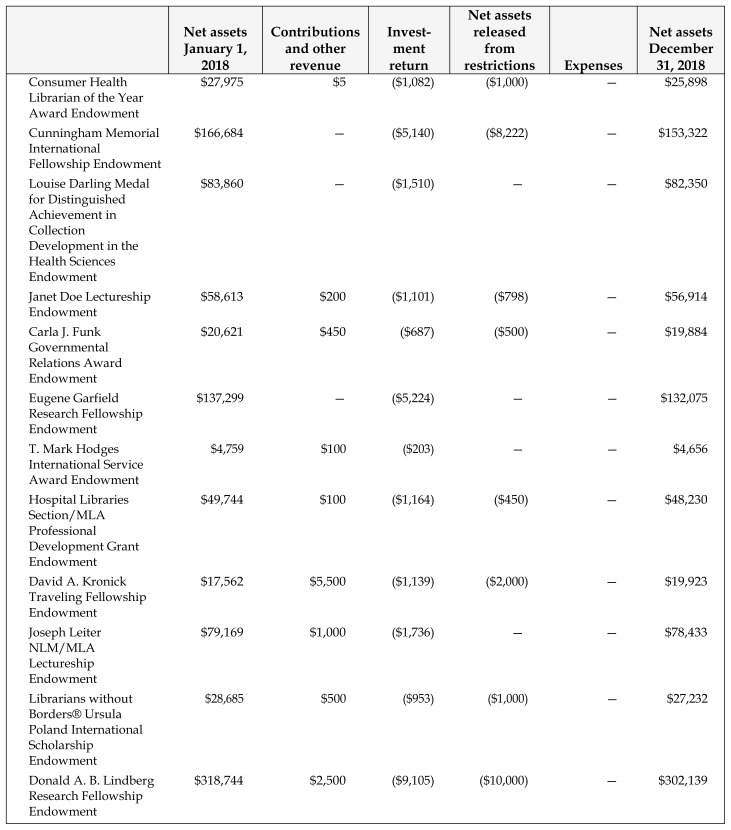
Medical Library Association schedule of changes in net assets by fund year ended December 31, 2018

	Net assets January 1, 2018	Contributions and other revenue	Investment return	Net assets released from restrictions	Expenses	Net assets December 31, 2018
**Net assets without donor restrictions**						
General operating	($166,924)	$2,993,293	—	—	($2,977,040)	($150,671)

Other funds:						
Association stabilization	$1,743,434	—	($54,674)	—	—	$1,688,760
Capital equipment	$2,228	—	—	—	—	$2,228
Special purpose	—	—	—	$96,883	($96,883)	—
Sections	$289,573	$48,612	—	—	($60,972)	$277,213

**Total net assets without donor restrictions**	$2,035,235	$48,612	($54,674)	$96,883	($157,855)	$1,968,201

**Net assets with donor restrictions**						

Ysabel Bertolucci MLA Annual Meeting Grant Endowment	$33,506	$500	($1,237)	($995)	—	$31,774
Estelle Brodman Award for the Academic Medical Librarian of the Year Endowment	$74,937	—	($1,232)	($500)	—	$73,205
Naomi C. Broering Hispanic Heritage Grant Endowment	$29,759	$50	($1,066)	($1,000)	—	$27,743
Lois Ann Colaianni Award for Excellence and Achievement in Hospital Librarianship Endowment	$26,960	$1,000	($642)	($500)	—	$26,818
Consumer Health Librarian of the Year Award Endowment	$27,975	$5	($1,082)	($1,000)	—	$25,898
Cunningham Memorial International Fellowship Endowment	$166,684	—	($5,140)	($8,222)	—	$153,322
Louise Darling Medal for Distinguished Achievement in Collection Development in the Health Sciences Endowment	$83,860	—	($1,510)	—	—	$82,350
Janet Doe Lectureship Endowment	$58,613	$200	($1,101)	($798)	—	$56,914
Carla J. Funk Governmental Relations Award Endowment	$20,621	$450	($687)	($500)	—	$19,884
Eugene Garfield Research Fellowship Endowment	$137,299	—	($5,224)	—	—	$132,075
T. Mark Hodges International Service Award Endowment	$4,759	$100	($203)	—	—	$4,656
Hospital Libraries Section/MLA Professional Development Grant Endowment	$49,744	$100	($1,164)	($450)	—	$48,230
David A. Kronick Traveling Fellowship Endowment	$17,562	$5,500	($1,139)	($2,000)	—	$19,923
Joseph Leiter NLM/MLA Lectureship Endowment	$79,169	$1,000	($1,736)	—	—	$78,433
Librarians without Borders® Ursula Poland International Scholarship Endowment	$28,685	$500	($953)	($1,000)	—	$27,232
Donald A. B. Lindberg Research Fellowship Endowment	$318,744	$2,500	($9,105)	($10,000)	—	$302,139
Majors/MLA Chapter Project of the Year Endowment	$19,563	—	($480)	($500)	—	$18,583
Lucretia W. McClure MLA Excellence in Education Award Endowment	$54,967	$725	($1,536)	($500)	—	$53,656
John P. McGovern Award Lectureship Endowment	$131,278	$500	($4,827)	—	—	$126,951
MLA Disaster Relief Fund	$6,354	—	—	—	—	$6,354
Scholarship Endowment	$264,817	$15,208	($12,650)	($13,460)	—	$253,915
Section Project of the Year Award Endowment	($142)	—	—	($500)	—	($642)
Shaping Our Future Endowment	$71,492	$600	($2,172)	—	—	$69,920
Special Purpose/Librarians without Borders®	$59,039	$135,000	—	($54,958)	—	$139,081
Special Purpose/MLA/NLM Spectrum Scholarships	$26,000	—	—	—	—	$26,000

**Total net assets with donor restrictions**	$1,792,245	$163,938	($54,886)	($96,883)	—	$1,804,414

**Total all net assets**	$3,660,556	$3,205,843	($109,560)	—	($3,134,895)	3,621,944

**BKD**, CPAs and Advisors, on behalf of the Medical Library Association, kelly.weaver@mci-group.com, Chicago, IL

